# Effects of high‐fat diet and treadmill running on bone marrow fat and bone properties in young male mice

**DOI:** 10.14814/phy2.71033

**Published:** 2026-07-29

**Authors:** Yuri Takamine, Noriko Ichinoseki‐Sekine, Takamasa Tsuzuki, Toshinori Yoshihara, Shuichi Machida, Hisashi Naito

**Affiliations:** ^1^ Graduate School of Health and Sports Science Juntendo University Chiba Japan; ^2^ Faculty of Liberal Arts The Open University of Japan Chiba Japan; ^3^ Faculty of Pharmacy Meijo University Aichi Japan

**Keywords:** bone formation, bone resorption, exercise, high‐fat diet, marrow fat

## Abstract

We investigated the effects of a high‐fat diet (HFD) and treadmill running on bone marrow fat (BMF) and bone properties in mice. Four‐week‐old male mice (*n* = 6/group) were assigned to four groups: standard diet (STD) or HFD (60% kcal from fat), under sedentary or exercise conditions. Mice performed treadmill running (6–18 m/min, 30 min/day, 5 days/week) for 16 weeks. At 20 weeks, body weight, adipose tissue mass, bone mineral density (BMD), bone strength, histomorphometry, and bone turnover markers were assessed. HFD significantly increased body weight and adipose tissue mass, whereas exercise attenuated HFD‐induced weight gain. Absolute BMD was unaffected by diet but lower in exercised mice. Weight‐adjusted BMD was lower in HFD‐fed mice and higher in exercised mice. BMF was elevated by HFD and was not reduced by exercise. The resorption marker was increased by HFD and was not attenuated by exercise, whereas the formation marker was elevated only in exercised mice. The ratio of bone resorption to bone formation markers was higher in HFD‐fed mice. No significant diet × exercise interactions were observed for turnover markers. In conclusion, HFD during growth increases BMF, whereas treadmill running attenuates HFD‐induced weight gain but does not prevent increases in BMF.

## 1 INTRODUCTION

Bone health is fundamental for maintaining quality of life throughout the lifespan. Bone is a complex organ composed of bone tissue and bone marrow. Recently, increasing attention has been paid to bone marrow fat (BMF), an ectopic fat depot located within the medullary cavity. Notably, accumulating evidence suggests that BMF is not a simple passive byproduct of aging or metabolic imbalance but may actively influence the bone microenvironment through adipokines and fatty acids secreted by adipocytes, thereby affecting bone cell activity and bone remodeling (Hardouin et al., 2014). BMF increases with age, as shown in cross‐sectional studies of men and women (Justesen et al., 2001). The same work demonstrated that age‐related BMF accumulation correlates with decreased bone volume. Moreover, inverse associations between BMF and bone mineral density (BMD) have been observed in both older adults and younger populations (Di Iorgi et al., 2010; Shen et al., 2012). Therefore, excessive BMF may be a detrimental factor for bone health across the lifespan.

Obesity has been associated with increased fat deposition in both muscle and bone (Slawik & Vidal‐Puig, 2006). Importantly, high‐fat diet (HFD) induces obesity and elevates BMF volume, negatively influencing bone turnover (Gkastaris et al., 2020). Studies in aged mice have shown that HFD increases bone adiposity, decreases BMD, and elevates pro‐inflammatory cytokine levels (Halade et al., 2010). Furthermore, HFD elevates bone resorption markers (Patsch et al., 2011), and diet‐induced obesity increases bone resorption in association with higher BMF (Halade et al., 2011).

Exercise is a well‐established strategy for enhancing bone mass and strength, particularly through mechanical loading. Nonetheless, its interaction with diet‐induced changes in BMF remains incompletely understood. Most existing studies have been conducted in aged or adult animals and human subjects, whereas little is known about the combined effects of HFD and exercise during the growth period—a critical window when approximately 90% of adult bone mass is acquired (Henry et al., 2004; Santos et al., 2017). Optimizing the bone microenvironment during this period may help prevent skeletal deterioration in later life.

Therefore, we aimed to investigate the effects of HFD and treadmill running exercise on BMF volume and bone properties in young male mice. We hypothesized that HFD would increase BMF and impair bone properties, while exercise might mitigate these adverse effects during the growth period.

## 2 MATERIALS AND METHODS

### 2.1 Animals and diet

Four‐week‐old male C57BL/6 mice (*n* = 24) were purchased from Japan SLC (Hamamatsu, Shizuoka, Japan). At 5 weeks of age, mice were randomly assigned to one of four groups: (1) standard diet + sedentary (STD + Sed, *n* = 6), (2) HFD + sedentary (HFD + Sed, *n* = 6), (3) standard diet + exercise (STD + Ex, *n* = 6), or (4) HFD + exercise (HFD + Ex, *n* = 6). All mice were housed for 16 weeks at 23°C ± 1°C under a 12‐h light/dark cycle. The STD (12.2% kcal from fat, CLEA Rodent Diet CE2) or HFD (60% kcal from fat, High Fat Diet 32) and water were provided ad libitum. All experimental procedures were approved by the Juntendo University Animal Care Committee (H27‐08).

### 2.2 Exercise intervention

Exercise was performed on a motorized treadmill at 0° incline for 30 min/day, 5 days/week, for 16 weeks. The running speed was set at 6 m/min during the first week and increased by 1 m/min each week until reaching 18 m/min, which was maintained during the final 4 weeks. The maximum speed was chosen based on the fastest pace the mice could reliably maintain. Each daily session began at a slower pace and was gradually accelerated to the target speed. Exercise was continued until 3 days before sample collection. All exercise sessions were conducted during the dark phase.

### 2.3 Sample collection and preparation

At 20 weeks of age, the mice were fasted overnight and then anesthetized with isoflurane. Blood samples were collected by exsanguination under deep anesthesia, and the mice were subsequently euthanized. Blood samples were left at room temperature for approximately 30 min and then centrifuged at 3000 rpm for 10 min to obtain serum. Serum samples were stored at −30°C until analysis. White adipose tissue was collected from the epididymal depot. The left femur was excised, cleaned of soft tissue, and immediately weighed and tested for maximum breaking force. The right lower leg was harvested and fixed in 4% paraformaldehyde (PFA; pH 7.2–7.4) at 4°C for approximately 48 h for subsequent micro‐computed tomography (micro‐CT) and histological analysis.

### 2.4 Three‐point bending test

Left femur midshaft mechanical strength was measured using a three‐point bending apparatus (Model TK‐252C; Muromachi Kikai Co., Ltd., Tokyo, Japan). Bones were placed on two supports spaced 8 mm apart, and a crosshead was applied to the metaphyseal region at a 2.0 mm/min displacement rate until fracture occurred.

### 2.5 Micro‐CT

Right femur BMD was measured using an animal X‐ray micro‐CT system (Latheta LCT‐200, Hitachi‐Aloka Medical, Tokyo, Japan) at 50 kV, 0.5 mA, with an axial field of view of 24 mm, voxel size of 48 × 96 μm, and slice thickness of 96 × 279 μm. After scanning, samples were stored in 70% ethanol at 4°C until paraffin embedding for histological assessment.

### 2.6 Histomorphometry

Right femurs were fixed in 4% paraformaldehyde at 4°C for approximately 48 h, rinsed in water, and dehydrated, and decalcified in 10% ethylenediaminetetraacetic acid (pH 7.4) at 4°C for approximately 48 h. Samples were embedded in paraffin, sectioned at 4 μm, and stained with hematoxylin and eosin (H&E). To ensure that equivalent anatomical regions were analyzed across samples, sections were selected based on growth plate morphology. One section per right femur was digitized using a virtual slide scanning system (VS120, Olympus, Tokyo, Japan). The analysis area was defined as the distal metaphysis, extending 5% of the bone length proximal to the growth plate and excluding the primary spongiosa. Six fields of view were analyzed from each section. All region selections and image analyses were performed by an investigator blinded to the experimental groups. Bone area and tissue area were quantified using KS400 software (Carl Zeiss, Oberkochen, Germany). Bone area/tissue area ratio was calculated as bone area divided by total tissue area. BMF was assessed as adipocyte area/tissue area (%), based on morphological identification. For BMF analysis, adipocyte areas were identified as empty spaces within the bone marrow cavity, excluding vascular structures containing red blood cells.

### 2.7 Serum biomarkers

Serum tartrate‐resistant acid phosphatase‐5b (TRAP‐5b; bone resorption marker) and procollagen type 1 N‐terminal propeptide (P1NP; bone formation marker) were measured using enzyme‐linked immunosorbent assay kits (TRAP‐5b: SB‐TR103, P1NP: AC‐33F1; Immunodiagnostic Systems) according to the manufacturer's instructions. The bone metabolism ratio was calculated by dividing bone resorption markers by bone formation markers.

### 2.8 Statistical analysis

Data are presented as mean ± standard deviation. Statistical significance was set at *p* < 0.05. A two‐way analysis of variance was used for the statistical analysis. If the significance level was reached, comparisons were performed using Bonferroni's post hoc test. Two‐way analysis of variance was performed using IBM SPSS Statistics software (Version 25.0; IBM Corp., Armonk, NY, USA). The distribution of P1NP data was assessed prior to analysis. Because the P1NP data were not normally distributed, potential outliers were identified using the interquartile range (IQR) method. Samples falling outside the IQR criterion were excluded from subsequent analyses.

## 3 RESULTS

### 3.1 Body weight and white adipose tissue weight

Body weight did not differ among the groups at baseline (4 weeks of age). By 20 weeks of age, body weight was significantly higher in the HFD groups than in the STD groups, indicating a significant main effect of diet (*p* < 0.05). Treadmill running also significantly reduced body weight, yielding a significant main effect of exercise (*p* < 0.05). No significant diet × exercise interaction was detected (*p* = 0.093) (Table 1). White adipose tissue weight was significantly greater in HFD groups than in the STD groups, indicating a significant main effect of diet (*p* < 0.05). Neither the main effect of exercise nor the diet × exercise interaction was significant (Table 1).

**TABLE 1:** Body weight and white adipose tissue weight.

	STD+Sed	STD+Ex	HFD+Sed	HFD+Ex
Body weight (g, 4 week)	17.0 ± 1.5	17.4 ± 1.2	17.3 ± 1.3	17.2 ± 1.4
Body weight (g, 20 week)	26.0 ± 1.7	24.0 ± 1.1	42.3 ± 2.8*	36.8 ± 3.3^†^
White adipose tissue (mg, 20 week)	328.9 ± 113.1	289.9 ± 75.9	1659.8 ± 399.2*	1761.3 ± 380.3*

*Note*: Results expressed as means ± standard deviation. A two‐way analysis of variance was performed.

Abbreviations: HFD + Ex, high‐fat diet + exercise; HFD + Sed, high‐fat diet + sedentary; STD + Ex, standard diet + exercise; STD + Sed, standard diet + sedentary.

* *p* < 0.05 versus STD + Sed.

^†^ *p* < 0.05 versus HFD + Sed.

### 3.2 Bone weight

**FIGURE 1:**
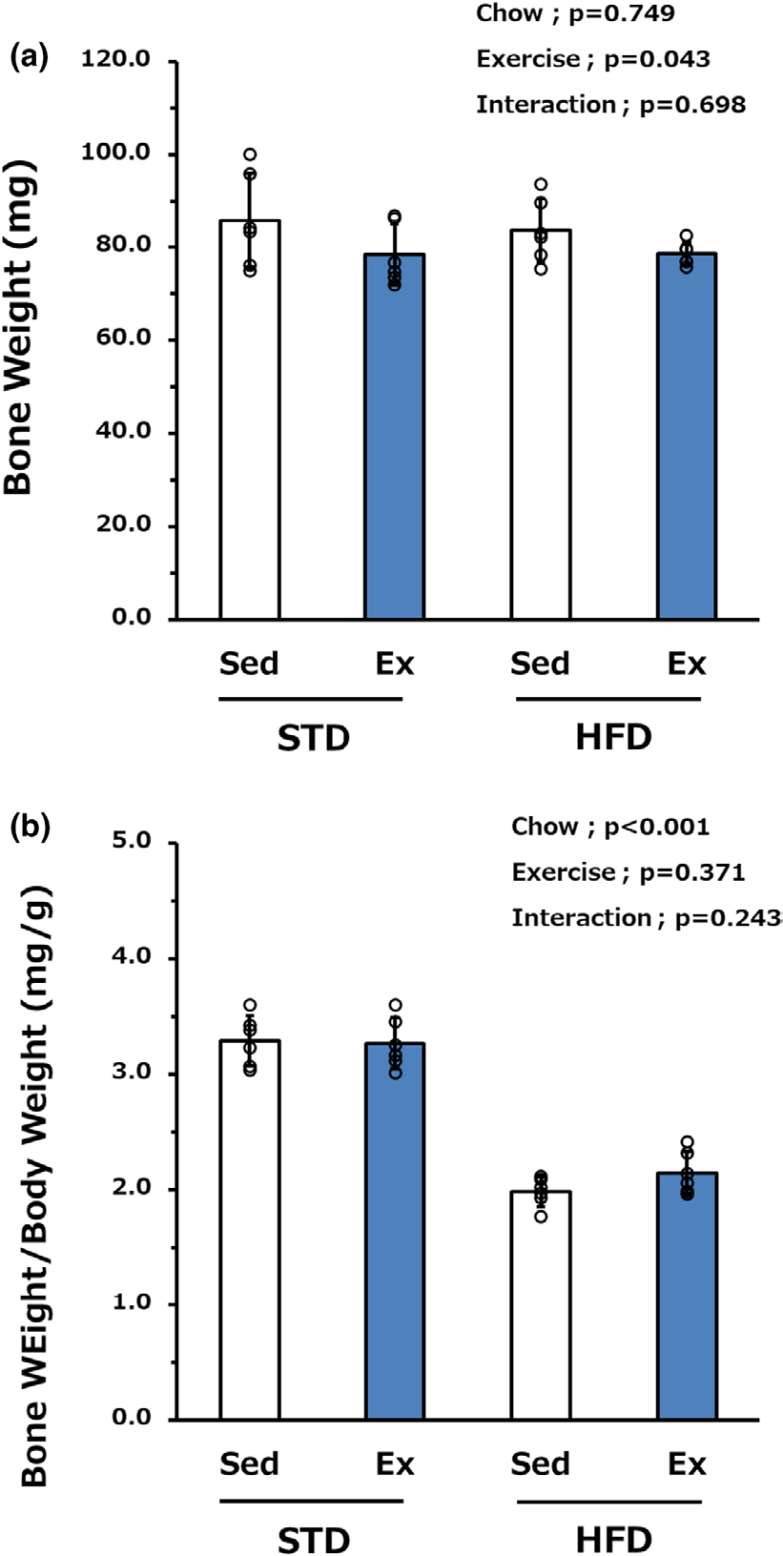
Femoral bone weight at 20 weeks of age. (a) Femoral bone weight and (b) Femoral bone weight normalized to body weight. Values are presented as mean ± SD. Statistical analyses were performed using two‐way ANOVA to evaluate the main effects of diet and exercise and their interaction. ANOVA, analysis of variance; Ex, exercise; HFD, high‐fat diet; SD, standard deviation; Sed, sedentary; STD, standard diet.

Femoral bone weight at 20 weeks of age did not differ significantly between the STD and HFD conditions. Two‐way ANOVA revealed a significant main effect of exercise on femoral bone weight (*p* < 0.05), whereas neither the main effect of diet nor the diet × exercise interaction was significant (Figure 1a). These findings indicate that the effect of exercise on femoral bone weight was similar under both dietary conditions. When bone weight was normalized to body weight, a significant main effect of diet was observed (*p* < 0.05). In contrast, neither a significant main effect of exercise nor a diet × exercise interaction was detected (Figure 1b).

### 3.3 Bone mechanical properties

**FIGURE 2:**
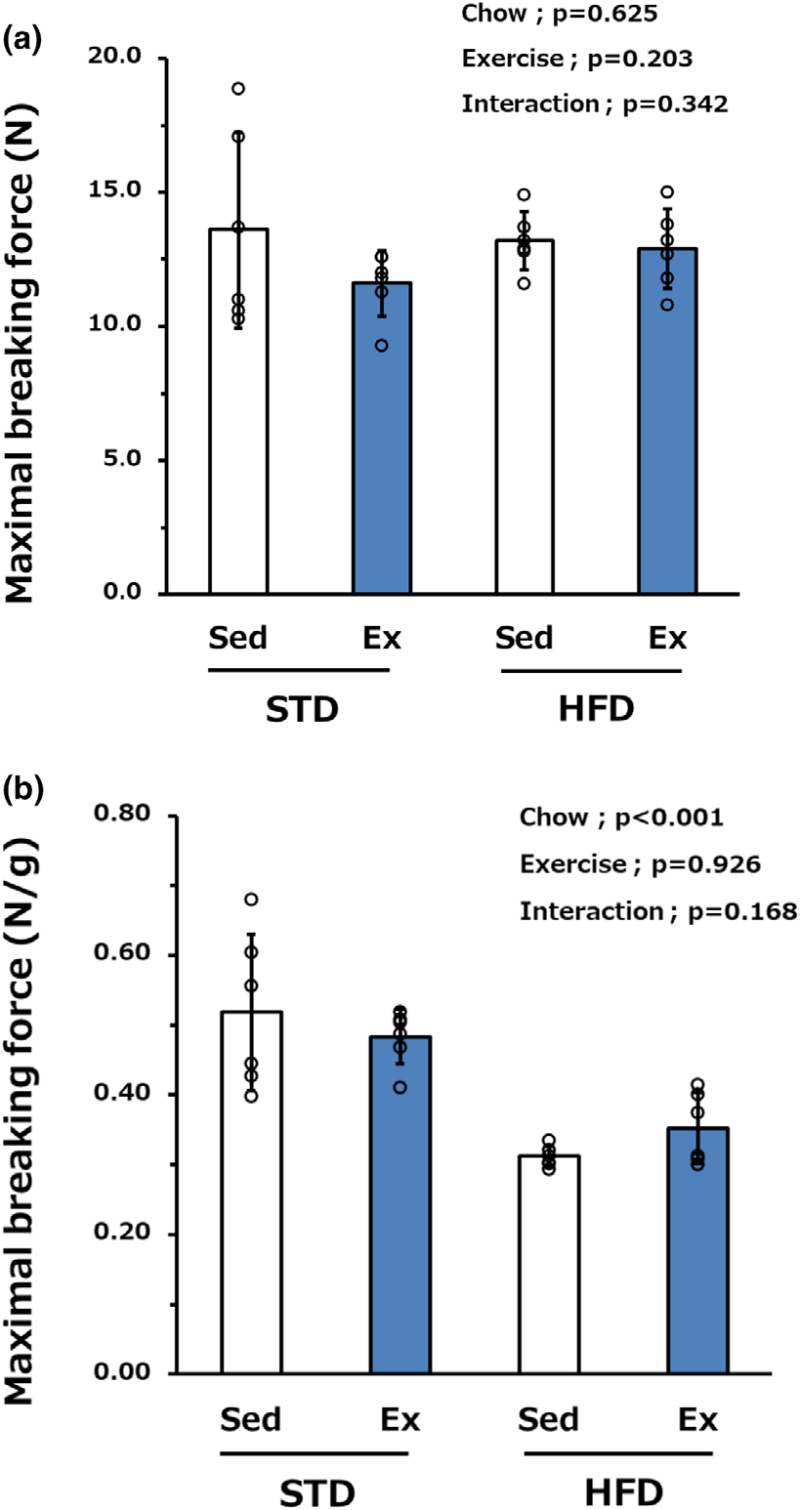
Femoral maximal breaking force at 20 weeks of age. (a) Femoral maximal breaking force and (b) Femoral maximal breaking force normalized to body weight. Values are presented as mean ± SD. Statistical analyses were performed using two‐way ANOVA to evaluate the main effects of diet and exercise and their interaction. ANOVA, analysis of variance; Ex, exercise; HFD, high‐fat diet; SD, standard deviation; Sed, sedentary; STD, standard diet.

Femoral maximal breaking force did not differ among the groups at 20 weeks of age, and no significant main effects of diet or exercise or a diet × exercise interaction were observed (Figure 2a). When femoral maximal breaking force was normalized to body weight, a significant main effect of diet emerged (*p* < 0.05, Figure 2b). In contrast, no significant main effect of exercise or diet × exercise interaction was detected (Figure 2a,b).

### 3.4 BMD (micro‐CT)

**FIGURE 3:**
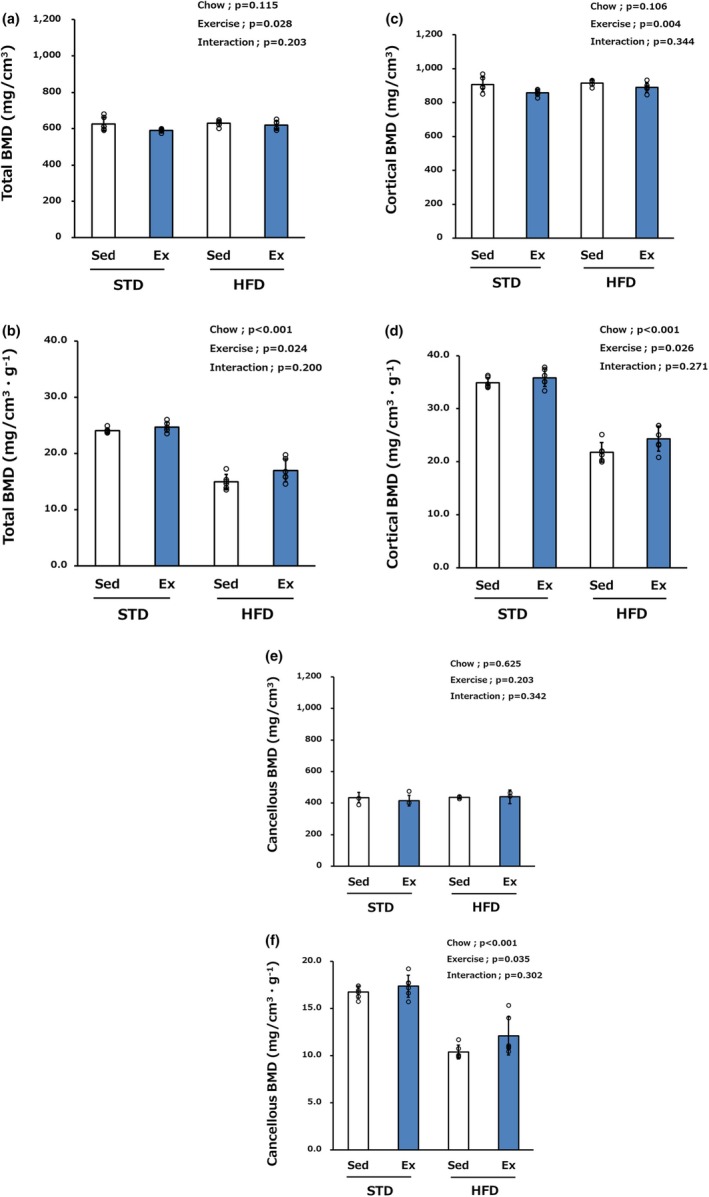
Bone mineral density (BMD) of the femur at 20 weeks of age. (a) Total BMD, (b) Total BMD normalized to body weight, (c) Cortical BMD, (d) Cortical BMD normalized to body weight, (e) Cancellous BMD, and (f) Cancellous BMD normalized to body weight. Values are presented as mean ± SD. Statistical analyses were performed using two‐way ANOVA to evaluate the main effects of diet and exercise and their interaction. ANOVA, analysis of variance; Ex, exercise; HFD, high‐fat diet; SD, standard deviation; Sed, sedentary; STD, standard diet.

At 20 weeks, total, cortical and cancellous BMD did not differ between the STD and HFD groups, indicating no main effect of diet. In contrast, significant main effects of exercise were observed for total and cortical BMD (*p* < 0.05; Figure 3a,c). When BMD values were normalized to body weight, significant main effects of both diet and exercise were observed for total, cortical, and cancellous BMD (*p* < 0.05, Figure 3b,d,f). No significant diet × exercise interactions were observed for any BMD parameter.

### 3.5 Bone histological properties

**FIGURE 4:**
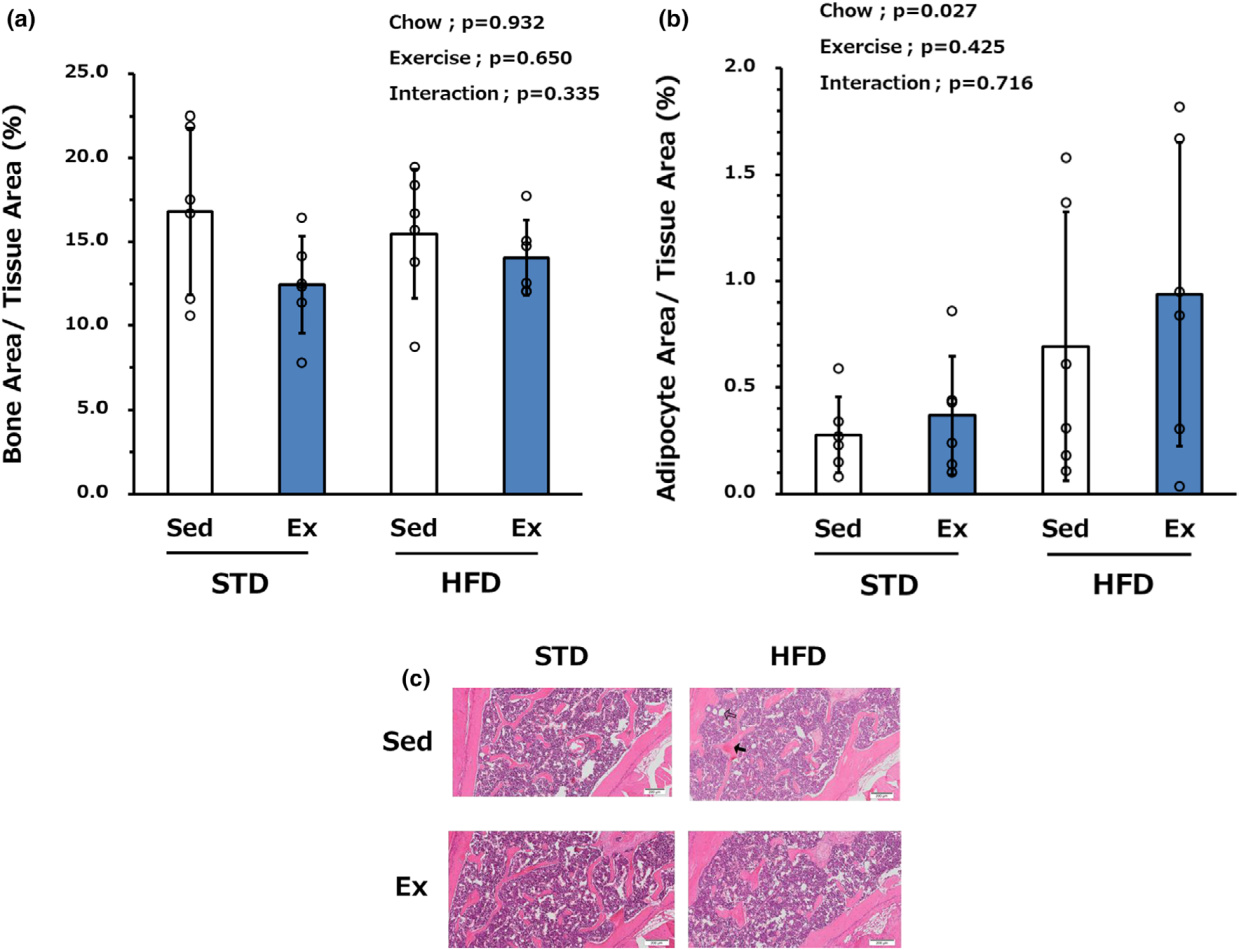
Histomorphometric analysis of the distal femur at 20 weeks of age. (a) Bone volume, (b) Bone marrow fat volume of the distal femur, and (c) Representative hematoxylin and eosin (H&E)‐stained femoral sections. Black arrow indicates bone tissue, whereas open arrow indicates fat. Values are presented as mean ± SD. Statistical analyses were performed using two‐way ANOVA to evaluate the main effects of diet and exercise and their interaction. ANOVA, analysis of variance; Ex, exercise; HFD, high‐fat diet; SD, standard deviation; Sed, sedentary; STD, standard diet.

Histological analysis of the distal femur revealed no significant differences in the two‐dimensional bone volume (B.Ar/T.Ar) among the groups. Accordingly, no significant main effects of diet or exercise were observed (Figure 4a). Bone marrow fat volume was significantly higher in the HFD than that in the STD groups, indicating a significant main effect of diet (*p* < 0.05; Figure 4b). In contrast, no significant main effect of exercise was observed. Furthermore, no significant diet × exercise interactions were detected for either bone volume or bone marrow fat volume (Figure 4a,b). Figure 4c shows a micrograph of bone volume and bone marrow fat. The black arrow indicates bone tissue, and the open arrow shows fat (C).

### 3.6 Bone turnover markers

**FIGURE 5:**
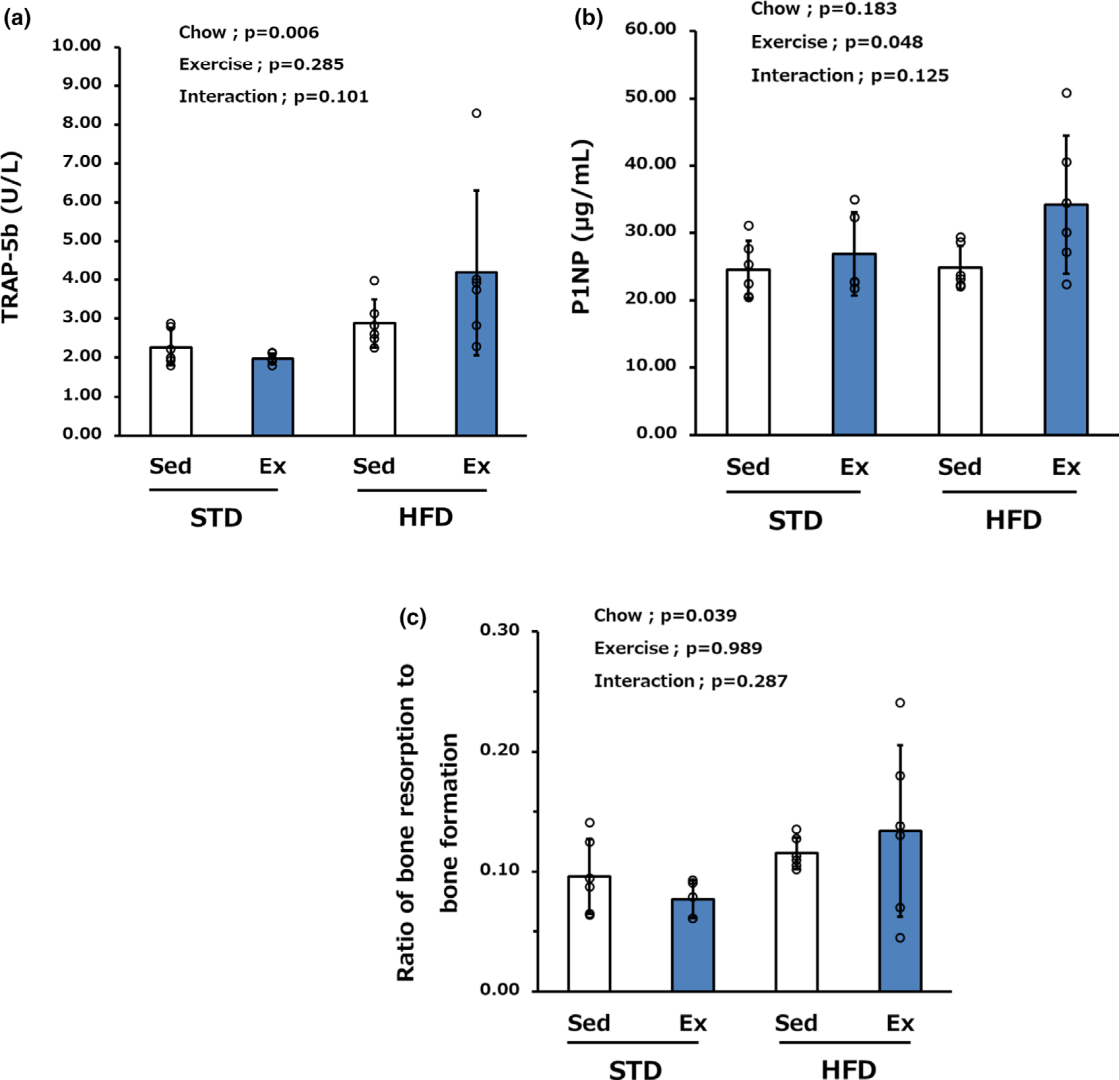
Serum bone turnover markers at 20 weeks of age. (a) Serum TRAP‐5b concentration, (b) Serum P1NP concentration, and (c) Serum TRAP‐5b/P1NP ratio. Values are presented as mean ± SD. The P1NP value of one mouse in the STD‐Ex group was excluded from the analysis as an outlier. Statistical analyses were performed using two‐way ANOVA to evaluate the main effects of diet and exercise and their interaction. ANOVA, analysis of variance; Ex, exercise; HFD, high‐fat diet; SD, standard deviation; Sed, sedentary; STD, standard diet; TRAP‐5b, serum tartrate‐resistant acid phosphatase‐5b.

Serum TRAP‐5b, a marker of bone resorption, was significantly higher in the HFD‐fed mice than in the STD‐fed mice, indicating a significant main effect of diet (*p* < 0.05; Figure 5a). The exercise did not significantly affect TRAP‐5b levels.

One sample in the exercise group (EX3; P1NP = 2.69 ng/mL) met the predefined IQR criterion for outliers and was excluded from the analysis. Serum P1NP, a marker of bone formation, was significantly elevated by exercise, indicating a significant main effect of exercise, whereas no main effect of diet was observed (Figure 5b).

The serum TRAP‐5b/P1NP ratio was significantly higher in the HFD‐fed mice, indicating a significant main effect of diet, whereas no main effect of exercise was detected (Figure 5c).

No significant diet × exercise interactions were observed for serum TRAP‐5b, serum P1NP, or the serum TRAP‐5b/P1NP ratio.

## 4 DISCUSSION

This study investigated the effects of HFD and treadmill running during the growth period on bone tissue and their combined effects. In young mice, 16 weeks of HFD significantly increased body weight and white adipose tissue mass, and these increases were markedly attenuated by treadmill running. HFD did not affect BMD, whereas treadmill running resulted in lower total and cortical BMD values. However, when BMD was normalized to body weight, significant main effects of both diet and exercise were observed for total, cortical, and cancellous BMD. These findings suggest that body weight substantially influences the interpretation of BMD during the growth period. In addition, BMF was increased by HFD but was not suppressed by concurrent treadmill running. The bone formation marker was unchanged by HFD, whereas the bone resorption marker was elevated by HFD. These findings indicate that the greater body mass associated with HFD may partially mask adverse effects on BMD when it is evaluated without adjustment for body weight.

Our previous work using OLETF rats, a model of type 2 diabetes, showed that voluntary wheel running before disease onset delayed diabetes development, reduced accumulation of BMF, and prevented its diabetes‐related increase, though bone strength was unaffected (Takamine et al., 2018). Similarly, Styner et al. (Styner et al., 2014) reported that voluntary running suppressed HFD‐induced accumulation of marrow adipose tissue and increased bone quantity in female mice, with marrow adipose tissue responding more rapidly to exercise without short‐term effects on bone quality, regardless of skeletal maturity. Conversely, the present study observed no suppression of HFD‐induced BMF by treadmill running, likely reflecting differences in exercise modality and total workload.

HFD may affect bone turnover via hormones as well as the bone microenvironment. PTH, calcitonin, and the active form of vitamin D 1,25(OH)2‐vitamin D3 [1,25(OH)2D3] regulate calcium homeostasis and control bone turnover. The effects of HFD on bone via these hormones vary among studies (Gkastaris et al., 2020; Liu et al., 2018; Ormanji et al., 2023). On the contrary, hormonal changes caused by exercise also affect bone turnover. Scott et al. have shown that high‐intensity exercise transiently increases blood PTH levels and β‐CTX, a bone resorption marker (Scott et al., 2011). In this study, mice were kept in a normal condition, the same as the control group, for 3 days after the exercise to avoid transient effects of the exercise, and samples were then taken to measure bone metabolism markers. Other markers, hormones that affect them, and blood calcium levels were not measured.

In recent years, accumulating evidence has suggested that physical activity and fitness during childhood and adolescence may influence health outcomes later in life (Hakala et al., 2019; Schmidt et al., 2016; Tait et al., 2022). For example, Hakala et al. reported that higher levels of physical activity from childhood to adulthood were associated with better reaction time in midlife (Hakala et al., 2019). Similarly, Shmidt et al. demonstrated that maintaining or improving physical fitness from youth into adulthood was associated with lower health risks later in life (Schmidt et al., 2016). These findings suggest that exercise during growth periods may have long‐term benefits extending beyond skeletal health. Although the present study focused on bone‐related outcomes, exercise during youth may contribute to lifelong health by suppressing obesity and promoting the functional development of multiple tissues and organ systems. Further studies should therefore examine whether benefits of exercise during growth extend beyond the musculoskeletal system and contribute to the coordinated development and long‐term maintenance of whole‐body physiological function.

This study has some limitations that warrant consideration. First, forced treadmill running was used in the present study to standardize exercise intensity and duration across animals. In contrast, voluntary wheel running is another commonly used exercise model in rodents that allows animals to run freely, often during their active nocturnal period. Although wheel running may better reflect natural activity patterns, the amount and intensity of exercise cannot be precisely controlled. Furthermore, rodents engaging in voluntary wheel running may accumulate substantially greater exercise volumes than the 30 min/day treadmill protocol used in the present study. Therefore, differences in exercise modality, exercise volume, and potential circadian influences should be considered when comparing the present findings with those from studies using voluntary wheel running. Second, the 4‐week‐old mice used in this study are in a period of rapid growth. HFD intake during growth may not have adverse effects on the bones of healthy mice. The HFD and the treadmill running in healthy young mice may have less detectable effects, at least in adulthood. Third, BMA quantification relied solely on two‐dimensional histology, which, while informative for morphology and localization, may lack accuracy compared to three‐dimensional assessments; thus, integrating volumetric imaging methods is recommended. Fourth, only male mice were included in this study, which limits the generalizability of our findings. Because bone metabolism is strongly influenced by sex hormones and exhibits well‐recognized sex‐specific characteristics, the skeletal responses to HFD and exercise may differ between males and females. Future studies including both sexes are therefore warranted to determine whether the present findings can be generalized across sexes. Fifth, although the sample size (*n* = 6 per group) is typical for rodent studies, it may have limited statistical power and increase the risk of type II errors. Therefore, subtle effects of HFD and exercise on bone turnover may not have been detected. In addition, skeletal growth and bone remodeling are highly active during this developmental stage, which may have influenced the responsiveness of bone turnover markers. Future studies with larger sample sizes are needed to confirm these findings. Finally, the present findings obtained in mice may not be directly generalizable to humans.

## 5 CONCLUSION

A HFD during growth increases BMF, and treadmill running exercise does not mitigate this effect in young male mice.

## AUTHOR CONTRIBUTIONS

**Yuri Takamine:** Conceptualization; data curation; formal analysis; funding acquisition; investigation; methodology; project administration; resources; validation; visualization. **Noriko Ichinoseki‐Sekine:** Formal analysis; methodology; resources; visualization. **Takamasa Tsuzuki:** Formal analysis; resources. **Toshinori Yoshihara:** Data curation; formal analysis; methodology; resources. **Shuichi Machida:** Supervision; validation. **Hisashi Naito:** Conceptualization; funding acquisition; project administration; resources; supervision.

## FUNDING INFORMATION

This research was funded by the Joint Research Program of Juntendo University, Faculty of Health and Sports Science. This study was also supported by the Institute of Health and Sports Science & Medicine, Juntendo University. This work was supported by the Research Encouragement Program of Juntendo University, Faculty of Health and Sports Science.

## CONFLICT OF INTEREST STATEMENT

The authors declare no conflicts of interest associated with this manuscript.

## ETHICS STATEMENT

All experimental procedures were approved by the Juntendo University Animal Care Committee (H27 – 08).

## Data Availability

Data supporting the findings of these studies are available upon request from the corresponding author.
